# Mindfulness in primary school children as a route to enhanced life satisfaction, positive outlook and effective emotion regulation

**DOI:** 10.1186/s40359-020-00428-y

**Published:** 2020-07-08

**Authors:** R. Amundsen, L. M. Riby, C. Hamilton, M. Hope, D. McGann

**Affiliations:** Department of Psychology, Northumbria University, Newcastle-upon-Tyne, NE1 8ST UK

**Keywords:** Mindfulness, Wellbeing, Schools, Cognitive reappraisal, Positive psychology

## Abstract

**Background:**

Mindfulness programmes as a potential avenue of enhancing pupil wellbeing are beginning to show great promise. However, research concerning the effectiveness of mindfulness training for primary aged school children (7–11 years of age) has been neglected.

**Methods:**

Building on methodological limitations of prior research, this study employed an active controlled design to assess the longer term wellbeing and emotion regulation outcomes after a 6 week mindfulness programme (*Living Mindfully Programme, UK*), for a group of school children aged between 9 and 10. The programme was delivered by class teachers as part of their normal curriculum entitlement. One hundred and eight children took part from across three schools in North East of England. Participants formed a treatment group (*n* = 64), active control (*n* = 19) and wait list control (*n* = 25). Self-report measures of wellbeing, mindfulness and emotion regulation were collected at pre and post training as well as at 3 months follow up.

**Results:**

Reliable findings, judged by medium to large effect sizes across both post intervention, follow-up and between both controls, demonstrated enhancement in a number of domains. Immediately after training and follow up, when compared with the wait list control, children who received mindfulness training showed significant improvements in mindfulness (*d* = .76 and .77), Positive Outlook (*d* = .55 and .64) and Life Satisfaction (*d* = .65 and 0.72). Even when compared to an active control, the effects remained although diminished reflecting the positive impact of the active control condition. Furthermore, a significant positive relationship was found between changes in mindfulness and changes in cognitive reappraisal.

**Conclusions:**

Taken together, this study provides preliminary evidence that the *Living Mindfully Primary Programme* is feasibly delivered by school staff, enjoyed by the children and may significantly improve particular components of wellbeing. Importantly, higher levels of mindfulness as a result of training may be related to effective emotional regulatory and cognitive reappraisal strategies.

## Background

Research suggests that quality of life in adulthood, social prospects, mental health and educational outcomes are influenced by emotional, psychological and social wellbeing during childhood [1]. It is thought that promoting resilience and protecting psychological wellbeing may be achieved through the presence of increased emotion regulation and coping skills [2]. Indeed, studies have shown healthy child development is dependent on effective emotion regulation [3]. Important to this study, wellbeing has been intrinsically linked to emotion regulation [4]. With this in mind, mindfulness meditation may be a vehicle for facilitating emotion regulation [5]. With mental health across schools in the UK being a priority [6], there is a drive to employ evidence based interventions which aim to promote pupil wellbeing. A handful of studies, see [7] for review, have suggested school based mindfulness training may enhance pupil wellbeing and even fewer studies have explored the mechanisms by which mindfulness may work. As such, this study endeavours to examine the impact of a mindfulness training programme on primary aged children’s subjective and psychological wellbeing and emotion regulation.

Being a multifaceted construct, the definition of wellbeing requires consideration. Two key definitions of wellbeing have been outlined in previous research. First, the *eudaemonic* perspective generally considers wellbeing in terms of the actualisation of human potentials. However, the second *hedonic* perspective considers components that are concerned with the immediate states of happiness and pleasure [8]. Both of these views of wellbeing are encapsulated in the terms *psychological and subjective wellbeing* respectively [9]. Psychological wellbeing is grounded in the *eudaemonic* perspective and relates to concepts such as personal relatedness, positive outlook, life purpose, mastery and personal growth [10]. In contrast, *subjective wellbeing* is seen to comprise satisfaction with life, the absence of negative mood and the presence of positive mood and may also refer to the affective and cognitive evaluations of one’s life [11]*.* It is generally considered that a combination of both psychological and subjective elements allows a fuller understanding of wellbeing [8]. Therefore, in the current study, a variety of indices of both psychological and subjective wellbeing and associated emotion regulation measures will be employed to capture the disparate nature of the construct and allowing a richer understanding of the impact of mindfulness training.

As an avenue for promoting wellbeing for their pupils, the past decade has seen a growth in the adoption of mindfulness-based programs within school curricula [2]. Mindfulness meditation aims to promote an awareness of the present moment with a non-judgmental focus of attention on thoughts, sensations, perceptions and feelings [5]. Mindfulness is considered to play a large role in freeing a person from automatic habits, thoughts and unhealthy behaviours. As such, it is thought to enhance self-regulation which in turn is related to the improvement of wellbeing [8]. A wealth of studies in adults have demonstrated how mindfulness can enhance satisfaction with life [12] as well as reduce stress and negative emotion [13].

Despite the positive results of mindfulness practice, the precise mechanisms leading to enhancement of wellbeing are somewhat unknown yet some research points to its potential to benefit emotion regulation [14]. Emotion regulation has been defined as the conscious and unconscious strategies one employs to maintain, increase or decrease components of an emotional response [15]. According to the process model [15], there are two main strategies of emotion regulation: suppression and reappraisal. That is, suppression is where emotions are inhibited, and reappraisal is where emotions are reinterpreted. A review of both experimental and individual difference studies suggests reappraisal of emotions is related to healthier wellbeing, affect and social functioning rather than to suppression [16]. For example, reappraisers are more likely to interpret stressful events in a more optimistic light and as a result tend to express more positive affect and less negative affect [17]. It is worth noting, however, that research tends to focus on down regulation of negative emotion yet in certain fields such as psychopathology, it has been suggested that suppression of positive emotion may be an adaptive strategy [18]. Through insights from clinical, neurobiological and psychological research, some authors propose mindfulness may be linked to the interplay of bottom up and top down emotion regulation mechanisms [13]. For example, Lutz et al. [19] found enhancement of attentional control mechanism subserved by multiple pre-frontal cortex regions in those individuals encouraged to use mindful strategies when processing negatively toned stimuli. Elsewhere, emotion regulation and attentional control have been highlighted to be the primary neurocognitive mechanisms involved in mindfulness [20]. Furthermore, some researchers have theorised that emotion regulation may serve as a potential mediator of the relationship between mindfulness and wellbeing [12]. Importantly, it is also thought that practicing mindfulness may lead to the improvement of emotion regulation through the specific mechanism of cognitive reappraisal [21]. It is also thought that mindfulness practice may lead to alterations in thought patterns and even self-management of behaviour by reducing emotional reactivity [22]. In spite of some empirically based findings and theoretical suggestions, further research is required to refine our understanding of how mindfulness practice is linked to emotion regulation mechanisms.

Although mindfulness practice programmes are traditionally designed for adults, programmes are beginning to emerge for use in schools with young people. Such programs include Paws b [23] and the focus of the present study, the Living Mindfully Primary Programme (LMPP). Both programmes are manualised and delivered by trained practitioners who can be the current class teacher or in-house school staff. Some have proposed that mindfulness training may benefit young people in a similar fashion to adults [24] but debate exists around the exact age at which mindfulness can be introduced most productively [25]. Certain distinct features of mindfulness training may make it particularly appropriate for use with children such as physical movement, concrete instructions and the use of short time periods [26]. Some authors have suggested that the use of analogy and metaphor in mindfulness programmes make it suitable for children aged eight and younger due to their affinity to metaphorical thinking [27]. Also, being a time of major developmental changes, preadolescence may be an ideal age for targeting wellbeing [28]. In contrast however, in line with a Piagetian framework, some have argued it may be essential for children to have reached the stage of formal operations at around aged 12 where hypothetical and abstract thinking are possible [29]. Evidently, the exact age at which mindfulness may be effectively introduced still remains debatable but strong arguments certainly suggest it may be suitable for primary aged children and hence the drive for such research endeavours.

Despite the optimal age being debatable, there are emerging findings which suggest mindfulness training can enhance a range of wellbeing outcomes. A very recent qualitative study which questioned experiences of teachers from the US and Australia revealed mindfulness may raise academic performance whilst also enhancing a child’s wellbeing [30]. A meta-analysis of school mindfulness training programmes for both younger children and adolescents conducted by Zenner et al. [31], reported a medium effect size among the domains of wellbeing such as coping, emotional problems and stress. Similarly, Zoogman et al. [32] in their meta-analytical review of young people aged 6–21 in both school and non-school settings revealed that mindfulness-based interventions improved outcomes including emotion regulation albeit with small effect sizes. Notwithstanding positive results, studies are not without their criticism. A systematic review of mindfulness training programmes with young people has highlighted methodological limitations such as a lack of controlled studies with follow up measures which is addressed in the current study [33]. Another more recent systematic review by Weare [7] recommended a greater number of randomised controlled trials (RCTs) and larger primary aged studies with longer follow ups and more replication. Despite this call for more larger RCTs, when working with schools where children are already allocated to set class groups, achieving ideal scientific conditions with random allocation is not always achievable [34]. Moreover, the use of random allocation can have its own limitations such as the diffusion of treatment. This transmission of knowledge by pupils or staff may in turn invalidate test results [34]. An additional criticism from another meta-analysis of mindfulness interventions with young people by Klingbeil et al. [35] was that most studies rarely included measures of the theoretical mechanisms by which mindfulness practice may improve outcomes such as self-reported mindfulness or emotion regulation. Appropriately, the analysis stressed the need for future studies to not just measure how effective mindfulness is for various purposes, but also how practice may actually work for young people. Finally, another limitation of many studies is their lack of consideration of program fidelity or treatment integrity [35]. In order to address this issue, the present study chose to evaluate a manualised program which is delivered by class teachers with appropriate training and a good standard of personal mindfulness practice. Furthermore, the adoption of a manualised programme with a reported ‘dosage’ of mindfulness training improves the ability to replicate findings [36]. Indeed, research conducted in this manner is essential if schools are to identify programs which are both cost effective and sustainable.

Even though primary aged children (aged 7–11 years) may be at an ideal developmental stage to receive mindfulness training to enhance wellbeing, research for this younger age group is under-represented and few studies have evaluated wellbeing as an independent construct in its own right. The review by Felver et al. [33] reported that across a large range of studies, the average age of participants was 12.3 and only eight of the 28 studies were with children of primary age. Furthermore, the majority of the studies rarely evaluated programmes delivered by the current class teacher and very few studies have been conducted in the UK.

One New Zealand based study which specifically investigated the effectiveness of mindfulness training in enhancing both psychological and subjective wellbeing of school children is that of Bernay, Graham, Devcich, Rix, and Rubie-Davies [37]. Self-reported measurements of wellbeing and mindfulness for 124 children aged 9–12 were taken at pre-intervention, post intervention and at three-month follow up using the Stirling Children’s Wellbeing Scale and Mindfulness Attention Scale modified for children respectively. Results indicated a positive effect over time for mindfulness and wellbeing between pre and post intervention measures with scores on wellbeing returning to baseline levels at follow up. Despite a number of strengths, which included three measurement points and a mixed methods design, a lack of a control group in this study limited the degree to which the influence of confounding variables on the results could be ruled out. With this in mind, a recent UK based study using a controlled design by Vickery and Dorjee [38] simultaneously examined the impact of the paws b mindfulness programme on subjective wellbeing, as well as meta-cognition, on a group of seventy-one children aged 7–9. Compared to the control group, the mindfulness training group demonstrated a significant reduction in negative affect at follow up. Teacher reports of meta-cognition scores were also enhanced. Finally, in contrast to the control group, positive changes in mindfulness scores were correlated with improvements in emotional awareness scores. This study had many strengths including a controlled design and the combination of evaluations from adults with child self-report measures. However, as noted by the authors themselves, a lack of the adoption of an active control partially reduces the validity of the conclusions. More rigorous designs which employ active controls are necessary for the control of non-specific factors which may include time, novelty, extra attention, group contact and even possible expectations of benefits. An even more recent study which employed a non-randomised design found that following a whole school mindfulness programme, adolescent participants saw a reduction in perceived stress [39]. Despite the impressive sample size of the study, it also failed to adopt an active control in the design.

Having reviewed the literature to date, it is evident there is a limited number of UK based research studies for primary school mindfulness programmes and their impact on children’s psychological and subjective wellbeing. Moreover, additional acceptability and feasibility data is needed to understand how particular programmes are suitable for delivery to primary aged children. As such, further empirical studies are critical to appropriately inform educationalist and wellbeing policy. With this in mind, the primary aim of the present study was to provide a quantitative evaluation of the potential longer-term impact of the Living Mindfully Programme on the psychological and subjective wellbeing of children aged 9–10. Not only are there very few studies on primary aged children, the study builds on the argument that pre-adolescence may be the ideal time to target wellbeing using mindfulness practice [28]. The study intended to achieve its aim by using a controlled design with a follow up; reporting on the frequency and dosage of the programme and also using a range of validated wellbeing measures to capture different components of wellbeing. This study, to the best of the researcher’s knowledge, is the first UK study to not only evaluate this particular programme, but to also build on methodological limitations of previous studies by including an active control into the study design. Due to emerging evidence that mindfulness may enhance wellbeing through its ability to improve emotion regulation skills, a secondary aim of this study was to investigate the relationship between emotion regulation strategies and mindfulness. A further aim was to also briefly evaluate the acceptability of a mindfulness programme as part of the children’s regular school curriculum. In doing so, the study addressed the following hypotheses:

- The mindfulness training group will show greater increases in levels of mindfulness, compared to both control groups at post training and at 3 months follow up.
- Furthermore, the mindfulness training group will show greater increases in psychological and subjective wellbeing, as well as emotion regulation, compared to both control groups at post training and at 3 months follow up.
- Changes in mindfulness, determined by change scores (pre scores subtracted from post intervention and follow-up scores), should be positively associated with changes in the psychological and subjective wellbeing, and emotion regulation measures.

## Method

### Design

A non-equivalent control group pre-test post-test mixed design, with both a wait list and active control was employed to evaluate the impact of a mindfulness programme (IV) on enhancing psychological and subjective wellbeing, emotion regulation skills (cognitive reappraisal) and mindfulness (DVs) in a group of primary school children. Self-report measures of DVs were taken at pre and post programme and at 3 months follow up to assess any potential lasting effects over time. The use of an active control, in addition to wait list control, aimed to determine whether any positive effects seen in the mindfulness group were attributable to mindfulness in itself and not to any additional non-specific factors of delivering an intervention designed to promote wellbeing in general.

### Participants

A total of 108, Year 5 children aged between 9 and 10 years old were recruited from three state primary schools in the North East of England. Schools were matched on basic socioeconomic characteristics and allocation to experimental groups was based on volunteer interest of staff who had been trained to deliver the Living Mindfully programme. Contact with eligible schools was made possible through liaison with a Local Authority Educational Psychology service who had facilitated staff mindfulness training across a number of their link schools. The first school to join the study (a) consisted of two separate Year 5 classes of children who were taught by their own permanent class teacher. One of those teachers had been trained to deliver the mindfulness sessions so that class was assigned to a mindfulness treatment group and the other to the wait list control. The second school (b) to join the study formed the active control group. The final school to join the study (c) formed a large treatment group composing of two separate Year 5 classes as it was their intention to deliver mindfulness to the whole cohort that term. See Table 1. below for detailed participant demographics.

**Table 1:** Participant demographic data

Variable	LMPP			WLC (a)	AC (b)	Total/Average
	(a)	(c)	(c)			
**Participants (N)**	17	26	21	25	19	108
**Age (years)**						
***M***	10.03	10.24	10.11	10.40	10.35	
***SD***	0.38	0.45	0.34	0.51	0.36	
**Gender (%)**						
**Female**	47%	61%	38%	48%	52%	49%
**Male**	53%	38%	62%	52%	48%	51%

*LMPP* Living Mindfully Primary Programme training group, *WLC* wait list control group’, *AC* active control group

### Measures

Using recommendations from the recent *Wellbeing Measurement Framework* [40], the present study utilised a range of validated self-report measures of wellbeing as well as self-report measures of emotion regulation and mindfulness. A brief acceptability measure was also administered to the larger of the two training groups (school c) to provide a short evaluation of the children’s experience of the programme. Where required, permission was obtained from the developers of any measures before use.

### Mindfulness: the child adolescent mindfulness measure (CAMM)

The CAMM [41] is a self-report measure which assesses the extent to which children have awareness and can observe and accept internal experiences in a non-avoidant and non-judgmental manner. It will be adopted to serve as an experimental manipulation check. Children are required to respond to ten items by deciding how well each item reflects their experience (e.g. At school, I walk from class to class without noticing what I’m doing). Items are reverse-scored and summed, with higher totals indicating greater mindfulness. The measure has indicated good.

concurrent validity and internal consistency (α = .87) as well as moderate negative correlations with somatic complaints, psychological inflexibility and externalising behaviour problems [41].

### Subjective wellbeing: the positive and negative affect scale for children (PANAS-C)

The PANAS-C [42] which was adapted from the original adult version [43], assesses both positive and negative affectivity using 27 items (12 positive affect (PA) and 15 negative affect (NA) items respectively). Children are asked to indicate the extent to which each statement (e.g. ‘delighted’ or ‘scared’) reflects their experience over the last few weeks. Each item is scored using a 5-point Likert scale ranging from 1 (not much at all) to 5 (a lot). Total scores for both PA and NA indicate the extent to which both domains of affectivity are experienced by the participant. The scale has demonstrated favourable psychometric properties: a study by Laurent et al. [42] reported alpha coefficients of .92 for the NA scale and .90 for the PA scale. It has also indicated good discriminant and convergent validity with existing measures of depression and anxiety [42, 44].

### Psychological and subjective wellbeing: Sterling Children’s wellbeing scale (SCWBS)

The SCWBS developed by Liddle and Carter [45] was one measure used to assess wellbeing. It is a 15 item positively worded self-report questionnaire designed to measure positive outlook (PO), which assesses *psychological wellbeing,* and positive emotional state (PES), which assesses *subjective wellbeing*. It aims to follow a contemporary positive psychology approach by addressing positive aspects of mental health in contrast to the deficit model. Positive outlook includes notions of clear thinking and optimism and positive emotional state aims to capture satisfying relationships and cheerfulness. Participants respond to items such as “I enjoy what each new day brings” and “I think lots of people care about me”. Each item is scored using a 5-point Likert scale ranging from 1 = never to 5 = all of the time and participants are asked to consider how each statement reflects their experience over the last few weeks. Both positive outlook and positive emotional state are assessed using 6 items each and higher scores indicates greater wellbeing. A social desirability subscale comprising of three items is also included in the measure to help identify any children who may be deliberately responding in a biased manner. Scores of 14–15 or 3 indicates scores which should be treated with caution in the analysis [45]. Finally, the scale indicates appropriate validity and reliability for the study. The test has been found to have good internal reliability, Cronbach’s alpha .83. and test-retest reliability (*n* = 701, *r* = .75). Construct validity was also indicated with significant positive correlations with the Warwick-Edinburgh Mental Wellbeing scale and the Dubois self-esteem scale (*r* = .75) [45].

### Subjective wellbeing: Student’s life satisfaction scale (SLSS)

Cognitive evaluations of subjective wellbeing were measured using the SLSS [46]. The SLSS is a global self-report measure of the life satisfaction concept. It is a seven-item questionnaire which asks children to respond to statements regarding overall life assessments not related to particular domains. Examples of items include “My life is just right” and “I wish I had a different kind of life”. Participants are asked to respond using a 6-point Likert scale ranging from strongly disagree (1) to strongly agree (6). Scores are summed and higher scores indicate greater satisfaction with the life. The scale demonstrates strong internal consistency α = 0.82 and moderate test re-test reliability (.74). It also demonstrates convergent validity with other wellbeing measures such as the Andrews-Withey Life Satisfaction Test [46].

### Emotion regulation

#### The emotion regulation questionnaire for children and adolescents (ERQ-CA)

The ERQ-CA is a valid age appropriate self-report measure of emotion regulation which is theoretically grounded in the emotion regulation theory of Gross [15]. Specifically, designed for children and adolescents, it is a revised version of the adult ERQ and assesses the two emotion regulation strategies of reappraisal (Reapp) and suppression (Supp). Children are asked to respond to a total of ten items using a Likert scale ranging from strongly disagree (1) to strongly agree (5). Six of the items are totalled to represent a score for reappraisal and the remaining four items for suppression. A psychometric evaluation by Gullone and Taffe [47] indicated a range of favourable properties including: good internal consistency (α = .83 for reappraisal sub scale and α = .75 for suppression); good construct and convergent validity as well as stability over a twelve-month period.

#### Acceptability measure

In order to assess acceptability, the study utilised a questionnaire which had been designed by the staff at Living Mindfully. The questions were designed for suitable use with primary aged children in order to briefly evaluate their experience of the training sessions. Children were asked to give their preferred answer to questions as a vote using a simple range of choices e.g. ‘Did you enjoy the mindfulness course?’, Vote: ‘Yes’, ‘It was okay’, or ‘No.’ This questionnaire was only administered to the large treatment group (*n* = 26 and *n* = 21) at the school denoted (c), post programme. Scores for each question are presented as percentages.

### Procedure

From initial meetings with school staff to final data collection, the study took place between November 2017 and July 2018. Prior to commencement, the study received ethical approval from the Department of Psychology, Northumbria University Ethics Committee. Before evaluation took place, informed consent was obtained from parents of participants. Participants were also asked whether they would like to take part in the study before each assessment session. Administration of the measures which assessed wellbeing, emotion regulation and mindfulness occurred at three different time points: prior to commencement of the mindfulness programme, immediately after and at 3 months follow up. The follow up assessment aimed to explore whether any effects observed were sustained over time. To reduce the effects of any potential demand characteristics and guard against any potential bias, children were informed using a standardised script that they were part of a wider study which was investigating wellbeing in school. The children were reminded: it was not a test; there were no ‘correct answers’; to respect each other’s privacy and answer the questions honestly. Furthermore, care was taken by staff to make no links between the mindfulness sessions and the wellbeing study. Parental letters and information sheets did not explicitly outline the exact details of the research question or hypotheses. All of the sessions were carried out by the researcher in the children’s regular classroom. During the sessions, standardised questionnaire instructions and test items were read aloud by the researcher to control for differences in reading ability. The class teacher and teaching assistants were also available to provide tailored support to children withEducational Needs. Each questionnaire session took approximately 25 min to administer. On the final session, participants received a small reward as a thank you and were debriefed verbally about the aims of the study.

#### Living mindfully primary Programme (treatment group)

The Living Mindfully Primary Programme (LMPP) is a mindfulness programme designed by certified mindfulness practitioners (www.livingmindfully.co.uk) who provide a range of mindfulness coaching and teaching sessions in the North of England. In order to be trained to deliver the LMPP, all school staff must initially complete the 8-week adult Mindfulness Based Stress Reduction course and have a minimum of 6 months personal practice. This extensive training and personal practice is required so that staff maintain fidelity to the approach. The programme is designed to encourage children to become more mindful of their present experiences in the classroom and become aware of automatic thought processes. It also incorporates breath and body-based practices which help explore the interplay between thoughts, emotions, physical sensations and relationships with others. Moreover, the programme utilises child friendly terminology which is referred to throughout all of the sessions to accommodate learning. The six programme workshop sessions include: ‘Know your mind’, ‘Know your body’, ‘Know your thoughts’, ‘Know your emotions’, ‘Know friendliness’ and ‘Know your Life’. The themes and sessions can be flexibly delivered by schools to suit their timetable demands but in the present study it was taught with six one-hour weekly sessions. Additionally, staff continued with short weekly mindfulness practice with the children in school between post and follow up.

#### Active control and wait list control

In order to control for non-specific factors associated with taught wellbeing programmes in school, a custom designed ‘wellbeing’ programme was planned by the researcher in conjunction with the class teacher. Being a current practicing primary teacher, the researcher was able to draw on pedagogical knowledge and experience to design a set of six one-hour sessions. The sessions were designed to match the Living Mindfully sessions in terms of nonspecific structural factors such as duration, adult contact and novelty [48]. In particular, the active control aimed to mirror the mindfulness programme in terms of design without specifically including any mindfulness techniques. The sessions drew upon a combination of PSHE curricular objectives, positive psychology approaches grounded in Seligman’s PERMA model [49] and the widely employed notion of growth Mindset [50]. Finally, the wait list control simply followed education as normal and provided a baseline comparison for all groups.

### Data treatment

Following the recommendation by van Breukelen [51] with non-randomised designs, two forms of analyses were carried out and contrasted to identify any inconsistencies in the effect of the mindfulness treatment. These included a 2(Time: post, follow-up) * 3 (Group: active control, wait list control, mindfulness) ANCOVA group comparison of the post treatment outcome measures with the pre-treatment measures as the co-variate, and a 2(Time: post change, follow-up change) * 3 (Group: control, wellbeing, mindfulness groups) ANOVA treatment with the change scores. The rationale for this recommendation was that a consistent treatment effect across both analyses would provide more reliable evidence for the findings. Partial eta-squared (ηp^2^) was also calculated and reported.

Where reliable significant effects were found, this was followed by one-way ANOVAs where differences between groups for each of the two time periods were examined. The first analysis was a validity check, which ensured that the mindfulness intervention had an effect on the level of mindfulness in the children.

Finally, further analyses using Pearson correlations assessed the relationship between pre to post and follow up changes in self-reported CAMM mindfulness scores and pre to post and follow up changes in all outcome measures.

## Results

Descriptive statistics for baseline, post-test and follow up scores may be seen in Table 2.

**Table 2:** Descriptive statistics for outcome measures at pre, post and follow up for each group

	Group					
**Measure**	**Wait List Control** (*n* = 25)		**Active Control** (*n* = 19)		**Living Mindfully** (*n* = 64)	
	***M***	*SD*	***M***	*SD*	***M***	*SD*
***CAMM***						
*Pre*	20.88	6.55	22.53	6.45	20.38	7.87
*Post*	18.88	9.08	23.47	5.65	24.75	6.85
*Follow Up*	18.68	7.82	23.63	5.12	25.30	7.65
**PANAS-C:PA**						
*Pre*	47.48	12.50	34.21	12.34	43.62	9.27
*Post*	47.44	10.90	37.53	9.97	47.21	8.23
*Follow Up*	47.88	10.37	38.32	9.07	47.16	8.50
**PANAS-C:NA**						
*Pre*	28.28	11.26	32.42	12.62	30.32	12.48
*Post*	27.44	9.41	27.39	11.18	26.31	10.67
*Follow Up*	27.36	9.37	27.68	11.27	24.20	9.15
**SCWBS:PeS**						
*Pre*	21.84	5.01	17.73	5.24	19.63	5.03
*Post*	21.96	5.80	19.15	5.23	22.14	4.61
*Follow Up*	21.80	5.68	19.00	5.03	22.40	4.68
**SCWBS: PO**						
*Pre*	22.76	4.77	19.58	4.47	20.58	5.35
*Post*	22.68	5.38	20.10	5.08	23.00	4.44
*Follow Up*	22.32	5.15	20.63	3.98	23.19	4.31
**SLSS**						
*Pre*	31.96	8.20	29.47	8.12	30.62	8.97
*Post*	29.88	9.50	29.74	10.69	33.34	6.95
*Follow Up*	29.64	8.73	30.11	9.13	34.32	7.38
**ERQ-CA: Reapp.**						
*Pre*	20.12	5.01	17.68	4.63	18.67	4.43
*Post*	20.44	5.90	18.26	4.60	20.64	4.60
*Follow Up*	19.88	5.75	18.79	3.89	19.59	4.67

*M* mean score, *SD* standard deviation, *CAMM* Child and Adolescent Mindfulness Measure, *PANAS-C PA and NA* Positive and Negative Affect Scale for Children, *SCWBS PeS/PO* Stirling Children’s Wellbeing Scale, Positive Emotional State and Positive Outlook subscales, *ERQ-CA* The Emotiona Regulation Questionnaire for Children and Adolescents Reappraisal Subscale

### The child adolescent mindfulness measure (CAMM)

This first analysis was a validity check, to ensure that the mindfulness intervention had an effect on the level of mindfulness in the children.

The 2*3 ANCOVA analysis revealed a non-significant effect of time, F (1, 104) = 2.436, *p* = .122, ηp^2^ = .023, a significant effect of group, F (2, 104) = 6.999, *p* = .001, ηp^2^ = .154, and a non-significant time * group interaction effect, F (2, 104) = .140, *p* = .869, ηp^2^ = .003, and a non-significant time * pre-treatment CAMM interaction F(1, 104) = 2.454, *p* = .120, ηp^2^ = .003. The 2*3 Change ANOVA analysis revealed a non-significant effect of time, F (1, 105) = .076, *p* = .783, ηp^2^ = .001, a significant effect of group, F (2, 105) = 6.999, *p* = .001, ηp^2^ = .118, and a non-significant interaction effect, F (2, 105) = .170, *p* = .844, ηp^2^ = .003. Consequently, a reliable effect associated with group was observed.

Subsequent one-way group ANOVAs for each of the two times revealed, post change, a significant effect of group, F (2, 105) = 6.151, *p* = .003, and for the follow up change, a significant effect of group, F (2, 105) = 6.313, *p* = .003. The effects sizes post change were *d = 0.76* (mindfulness vs. control), *d = 0.44* (mindfulness vs. active control) and for follow up change were d = 0.77 (mindfulness vs. control), d = 0.43 (mindfulness vs. active control). Finally, the internal consistency of the CAMM was acceptable at pre, post and follow up, Cronbach’s a = .75, .75 and .78 respectively.

#### Subjective wellbeing: the positive and negative affect scale for children (PANAS-C)

##### PANAS – positive affect

The 2*3 ANCOVA analysis revealed a non-significant effect of time, F (1, 104) = 3.028, *p* = .085, ηp^2^ = .028, a non-significant effect of group, F (2, 104) = 2.932, *p* = .058, ηp^2^ = .053, and a non-significant time * group interaction effect, F (2, 104) = 0.187, *p* = .829, ηp^2^ = .004, and a non-significant time * pre-treatment CAMM interaction F(1, 104) = 2.676, *p* = .105, ηp^2^ = .025. The 2*3 Change ANOVA analysis revealed a non-significant effect of time, F (1, 105) = .365, *p* = .547, ηp^2^ = .003, a non-significant effect of group, F (2, 105) = 2.172, *p* = .119, ηp^2^ = .040, and a non-significant interaction effect, F (2, 105) = .180, *p* = .835, ηp^2^ = .003. The effect sizes post change were *d* = 0.48 (mindfulness vs. control), *d* = 0.04 (mindfulness vs. active control) and for follow up change were *d* = 0.36 (mindfulness vs. control), *d* = − 0.07 (mindfulness vs. active control). The internal consistency of the 12 items of the PA scale were good at pre, post and follow up, Cronbach’s α = .86, .85 and .84 respectively.

### PANAS- negative affect

The 2*3 ANCOVA analysis revealed a non-significant effect of time, F (1, 104) = 2.322, *p* = .131, ηp^2^ = .022, a non-significant effect of group, F (2, 104) = 1.767, *p* = .176, ηp^2^ = .033, and a non-significant time * group interaction effect, F (2, 104) = 1.810, *p* = .169, ηp^2^ = .034, and a significant time * pre-treatment CAMM interaction F(1, 104) = 4.423, *p* = .038, ηp^2^ = .041. The 2*3 Change ANOVA analysis revealed a non-significant effect of time, F (1, 105) = .872, *p* = .353, ηp^2^ = .008, a non-significant effect of group, F (2, 105) = 1.909, *p* = .153, ηp^2^ = .035, and a non-significant interaction effect, F (2, 105) = 1.720, *p* = .184, ηp^2^ = .032. The effects sizes post change were *d* = − 0.32 (mindfulness vs. control), *d* = 0.11 (mindfulness vs. active control) and for follow up change were *d* = − 0.48 (mindfulness vs. control), *d* = − 0.14 (mindfulness vs. active control). The internal consistency of the NA scale was good at pre, post and follow up, Cronbach’s α = .85, .86 and .86 respectively.

#### Psychological and subjective wellbeing: Sterling Children’s wellbeing scale (SCWBS)

##### Positive emotional state – SCWBS: PES

The 2*3 ANCOVA analysis revealed a non-significant effect of time, F (1, 104) = 0.090, *p* = .765, ηp^2^ = .001, a marginally non-significant effect of group, F (2, 104) = 2.956, *p* = .056, ηp^2^ = .054, and a non-significant time * group interaction effect, F (2, 104) = 0.165, *p* = .848, ηp^2^ = .001, and a non-significant time * pre-treatment PES interaction F(1, 104) = 0.115, *p* = .735, ηp^2^ = .001. The 2*3 Change ANOVA analysis revealed a non-significant effect of time, F (1, 105) = .008, *p* = .927, ηp^2^ = .000, a marginally non-significant effect of group, F (2, 105) = 3.003, *p* = .054, ηp^2^ = .054, and a non-significant interaction effect, F (2, 105) = .171, *p* = .843, ηp^2^ = .003. The effects sizes post change were *d* = 0.49 (mindfulness vs. control), *d* = 0.22 (mindfulness vs. active control) and for follow up change were *d* = 0.54 (mindfulness vs. control), *d* = 0.28 (mindfulness vs. active control). The internal consistency of the PeS subscale scale was good at pre, post and follow up, Cronbach’s α = .82, .86 and .86 respectively.

##### Positive outlook – SCWBS: PO

The 2*3 ANCOVA analysis revealed a non-significant effect of time, F (1, 104) = 1.933, *p* = .167, ηp^2^ = .018, a significant effect of group, F (2, 104) = 4.827, *p* = .010, ηp^2^ = .085, and a non-significant time * group interaction effect, F (2, 104) = 0.209, *p* = .812, ηp^2^ = .004 and a non-significant time * pre-treatment PO interaction F(1, 104) = 1.823, *p* = .180, ηp^2^ = .017. The 2*3 Change ANOVA analysis revealed a non-significant effect of time, F (1, 105) = .111, *p* = .740, ηp^2^ = .001, a significant effect of group, F (2, 105) = 4.784, *p* = .010, ηp^2^ = .084, and a non-significant interaction effect, F (2, 105) = .439, *p* = .646, ηp^2^ = .008. Consequently, a reliable effect associated with group was observed.

Subsequent one-way ANOVAs for each of the two times revealed, post change, a significant effect of group, F (2, 105) = 3.788, *p* = .026. Note that Table 4 below indicates that the post change difference in PO was significantly correlated with change in CAMMM. For the follow up change, a significant effect of group, F (2, 105) = 4.526, *p* = .013. Table 4 indicates that this change was significantly associated with the change in CAMM. The effects sizes post change were *d* = 0.55 (mindfulness vs. control), *d* = 0.42 (mindfulness vs. active control) and for follow up change were *d* = 0.64 (mindfulness vs. control), *d* = 0.33 (mindfulness vs. active control). Finally, the internal consistency of the PO subscale was good at pre, post and follow up, Cronbach’s α = .81, .82 and .80 respectively.

##### *Sterling Children’s Wellbeing Scale (SCWBS)* Total

The 2*3 ANCOVA analysis revealed a non-significant effect of time, F (1, 104) = 0.824, *p* = .366, ηp^2^ = .008, a significant effect of group, F (2, 104) = 4.496, *p* = .013, ηp^2^ = .080, and a non-significant time * group interaction effect, F (2, 104) = 0.130, *p* = .879, ηp^2^ = .002, and a non-significant time * pre-treatment SCWBS interaction F(1, 104) = 0.815, *p* = .369, ηp^2^ = .008. The 2*3 Change ANOVA analysis revealed a non-significant effect of time, F (1, 105) = .019, *p* = .892, ηp^2^ = .000, a significant effect of group, F (2, 105) = 4.747, *p* = .011, ηp^2^ = .083, and a non-significant interaction effect, F (2, 105) = .250, *p* = .779, ηp^2^ = .005. Consequently, a reliable effect associated with group was observed.

However, Levene’s test indicates that the error variance of the dependent variable was not equal across groups in SCWBS change ANOVA analysis.

Subsequent one-way ANOVAs for each of the two times revealed, post change, a significant effect of group, F (2, 105) = 3.702, *p* = .028. Table 4 indicates that this post intervention change was significantly associated with the change in CAMM. For the follow up change, a significant effect of group, F (2, 105) = 4.659, *p* = .012. Table 4 indicates that this Follow Up change was significantly associated with the change in CAMM. The effects sizes post change were *d* = 0.57 (mindfulness vs. control), *d* = 0.35 (mindfulness vs. active control) and for follow up change were *d* = 0.65 (mindfulness vs. control), *d* = 0.34 (mindfulness vs. active control).

#### Subjective wellbeing: Student’s life satisfaction scale (SLSS)

The 2*3 ANCOVA analysis revealed a significant effect of time, F (1, 104) = 5.137, *p* = .025, ηp^2^ = .047, a significant effect of group, F (2, 104) = 6.258, *p* = .003, ηp^2^ = .107, and a non-significant time * group interaction effect, F (2, 104) = 0.608, *p* = .546, ηp^2^ = .012, and a significant time * pre-treatment SLSS interaction F(1, 104) = 4.543, *p* = .035, ηp^2^ = .042. The 2*3 Change ANOVA analysis revealed a non-significant effect of time, F (1, 105) = 0.583, *p* = .447, ηp^2^ = .006, a significant effect of group, F (2, 105) = 5.446, *p* = .006, ηp^2^ = .094, and a non-significant interaction effect, F (2, 105) = .716 *p* = .491, ηp^2^ = .013. Consequently, a reliable effect associated with group was observed.

Subsequent one-way Group ANOVAs for each of the two times revealed, post change, a significant effect of group, F (2, 105) = 4.366, *p* = .015. Table 4 indicates that this Post Intervention change was significantly associated with the change in CAMM. For the follow up change, a significant effect of group, F (2, 105) = 5.629, *p* = .005. Table 4 indicates that this Follow Up change was significantly associated with the change in CAMM. The effects sizes post change were *d* = 0.65 (mindfulness vs. control), *d* = 0.35 (mindfulness vs. active control) and for follow up change were *d* = 0.72 (mindfulness vs. control), *d* = 0.38(mindfulness vs. active control). Finally, the internal consistency of the SLSS was good at pre, post and follow up, Cronbach’s α = .86, .89 and .89 respectively.

#### The emotion regulation questionnaire for children and adolescents (ERQ-CA)

##### Reappraisal – ERQ-CA:Reapp

The 2*3 ANCOVA analysis revealed a non-significant effect of time, F (1, 104) = 0.021, *p* = .885, ηp^2^ = .000, a non- significant effect of group, F (2, 104) = 0.741, *p* = .479, ηp^2^ = .014, and a non-significant time * group interaction effect, F (2, 104) = 1.165, *p* = .879, ηp^2^ = .022, and a non-significant time * pre-treatment ERQ_Re interaction F(1, 104) = 0.146, *p* = .703, ηp^2^ = .001. The 2*3 Change ANOVA analysis revealed a non-significant effect of time, F (1, 105) = .717, *p* = .399, ηp^2^ = .007, a nonsignificant effect of group, F (2, 105) = 0.960, *p* = .386, ηp^2^ = .018, and a non-significant interaction effect, F (2, 105) = 1.215, *p* = .301, ηp^2^ = .023. The effects sizes post change were *d* = 0.34 (mindfulness vs. control), *d* = 0.28 (mindfulness vs. active control) and for follow up change were *d* = 0.24 (mindfulness vs. control), *d* = − 0.04 (mindfulness vs. active control). The internal consistency of the ERQ-CA Reappraisal scale was in the questionable to good range at pre, post and follow up, Cronbach’s α = .68, .83 and .79 respectively.

##### Suppression - ERQ-CA:Supp

The 2*3 ANCOVA analysis revealed a non-significant effect of time, F (1, 104) = 1.851, *p* = .177, ηp^2^ = .017, a non- significant effect of group, F (2, 104) = 0.176, *p* = .839, ηp^2^ = .003, and a significant time * group interaction effect, F (2, 104) = 3.202, *p* = .045, ηp^2^ = .058, and a non-significant time * pre-treatment ERQ Supp interaction F(1, 104) = 3.093, *p* = .082, ηp^2^ = .029. The 2*3 Change ANOVA analysis revealed a non-significant effect of time, F (1, 105) = .987, *p* = .323, ηp^2^ = .009, a nonsignificant effect of group, F (2, 105) = 0.109, *p* = .897, ηp^2^ = .002, and a non-significant interaction effect, F (2, 105) = 2.664, *p* = .074, ηp^2^ = .048. The effects sizes post change were *d* = 0.24 (mindfulness vs. control), *d* = 0.21 (mindfulness vs. active control) and for follow up change were *d* = − 0.05 (mindfulness vs. control), *d* = −-0.16 (mindfulness vs. active control).

#### The emotion regulation questionnaire for children and adolescents (ERQ-CA) Total

The 2*3 ANCOVA analysis revealed a non-significant effect of time, F (1, 104) = 0.423, *p* = .517, ηp^2^ = .004, a non- significant effect of group, F (2, 104) = 0.223, *p* = .800, and a significant time * group interaction effect, F (2, 104) = 3.688, *p* = .028, ηp^2^ = .066, and a non-significant time * pre-treatment ERQ_Sum interaction F(1, 104) = 0.882, *p* = .350, ηp^2^ = .008. The 2*3 Change ANOVA analysis revealed a non-significant effect of time, F (1, 105) = 1.987, *p* = .171, ηp^2^ = .018, a nonsignificant effect of group, F (2, 105) = .934, *p* = .396, ηp^2^ = .017,and a significant interaction effect, F (2, 105) = 3.569, *p* = .032, ηp^2^ = .064. Consequently, a reliable effect associated with the time * group interaction was observed.

Subsequent one-way ANOVAs for each of the two times revealed, post change, a nonsignificant effect of group, F (2, 105) = 2.009, *p* = .139. Table 4 indicates that this Post Intervention change was significantly associated with the change in CAMM. For the follow up change, a non-significant effect of group, F (2, 105) = .530, *p* = .590. Table 4 indicates that this change was not significantly associated with the change in CAMM. The effects sizes post change were *d* = 0.41 (mindfulness vs. control), *d* = 0.31 (mindfulness vs. active control) and for follow up change were *d* = 0.17 (mindfulness vs. control), *d* = − 0.13 (mindfulness vs. active control).

Table 3 above summarises the pattern of effect sizes associated with group differences in the CAMM and the other outcome measures.

**Table 3:** Summary of effect sizes for change scores at post and follow up (Cohen’s d)

Measure	Post score change	Follow up score change
**CAMM**		
Living Mindfully vs WLC	0.76	0.77
Living Mindfully vs AC	0.44	0.43
**SCWBS:PO**		
Living Mindfully vs WLC	0.55	0.64
Living Mindfully vs AC	0.42	0.33
**SLSS**		
Living Mindfully vs WLC	0.65	0.72
Living Mindfully vs AC	0.35	0.38

Table 4 below identifies the correlations between the outcomes change measures at post intervention and follow up points of time. Generally, changes in the CAMM measure were significantly associated with changes in the the outcome measures.

**Table 4:**
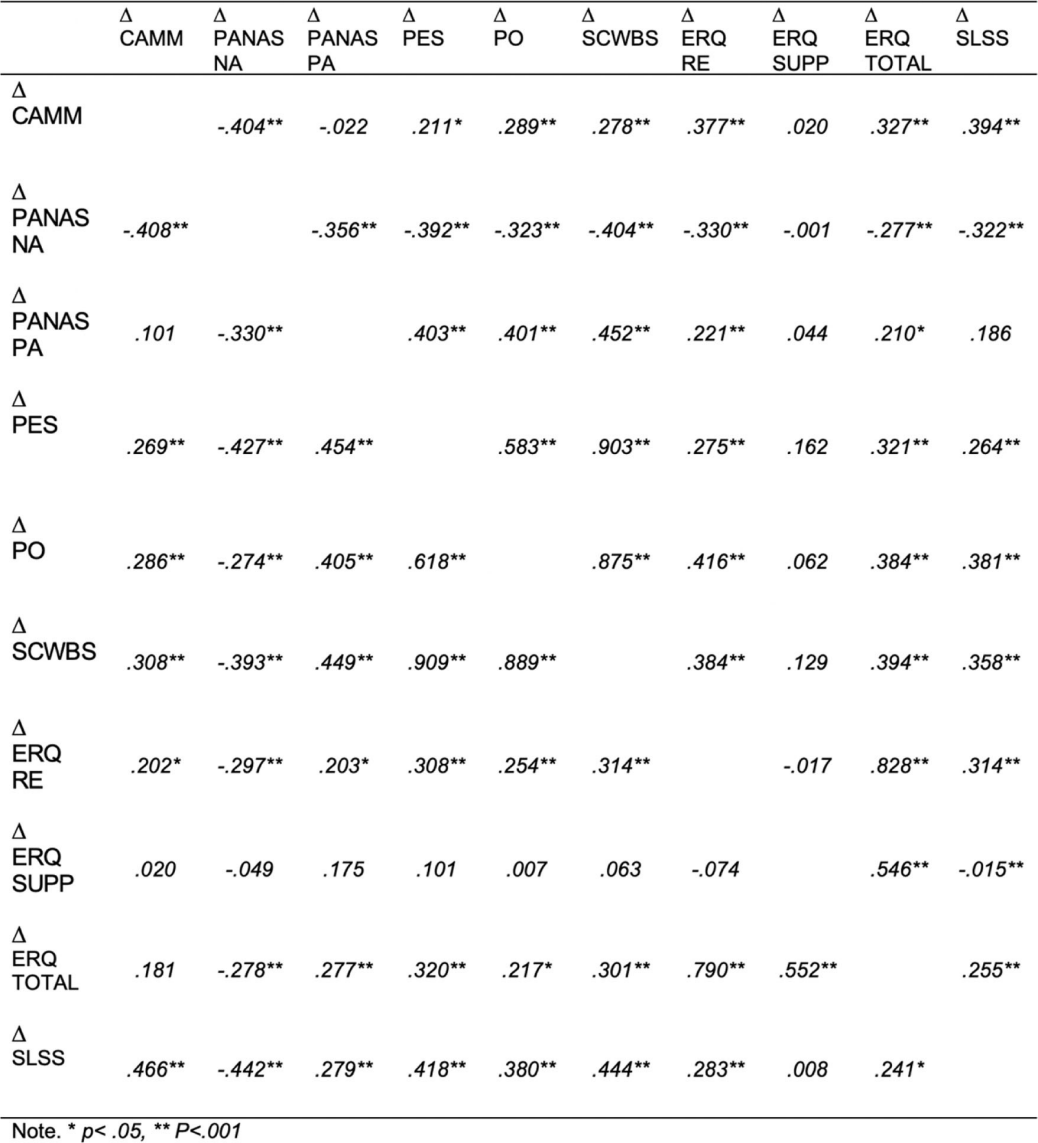
Correlations between the change scores of the variables. Above the diagonal, the change scores measured at Post intervention, below the diagonal the changes scores measured at Follow Up

##### Acceptability

The majority of the large treatment group (*n* = 51) reported that they enjoyed the mindfulness sessions (67%), 31% reported it was ‘okay’ and only 2% reported that they did not enjoy them. In terms of content of the course, 18% preferred the practices, 25% preferred the film clip element; 37% preferred the activities and 27% preferred the discussion aspect of the sessions. When asked if they ‘feel they are now living with more friendliness towards themselves and others’, 55% answered ‘yes’, 43% reported that they did not know and 2% answered ‘no’. When asked if they thought mindfulness was helpful to learn, the vast majority answered ‘yes’ (73%), 25% were ‘not sure’ and only 2% responded ‘no’. Finally, the children were asked to consider how mindfulness may help them in the future. The four most common themes which emerged were ‘friendships’, ‘stress relief’, ‘self-belief’ and ‘confidence’.

##### Relationship between mindfulness and the other outcome measures

Table 4 above indicates that changes in mindfulness, as measured by CAMM, was significantly associated with changes in psychological wellbeing, subjective wellbeing and emotion regulation. This was evident with pre-post changes and pre-follow up changes.

In summary, as expected the treatment group demonstrated significant changes in mindfulness, as measured by the CAMM, in comparison to the wait list control and active control groups. The pattern of results indicated group differences in psychological and subjective wellbeing.

## Discussion

The present study aimed to evaluate the acceptability and the impact of a mindfulness programme on the psychological and subjective wellbeing and emotion regulation with a group of primary school children aged between 9 and 10. Findings show that the programme was not only feasibly delivered by school staff, but also enjoyed and valued by the majority of the children. In support of the primary hypothesis, findings suggest that mindfulness training significantly increased the self-reported wellbeing domains of Positive Outlook and Life Satisfaction with medium and small effect sizes when compared to the wait list and active control groups respectively. Moreover, increases in pupil wellbeing were positively associated with significant increases in levels of self-reported mindfulness. Note that the intervention did not significantly impact upon emotion regulation.

The wellbeing findings are in agreement with previous studies which have reported the association between positive emotional states and mindfulness [28, 52]. The results also build upon findings from a non-controlled study which found a school-based mindfulness programme may lead to improvements in wellbeing as measured by the SCWBS [37] (but note that Levene’s measure indicates that this analysis’ parametric assumption was compromised). The Bernay et al. study also found positive correlations between change in PO and mindfulness scores which is also in line with findings of the present study. To the researcher’s knowledge, no previous studies have examined changes in the specific construct of Life Satisfaction in response to mindfulness training in schools. However, the current findings are congruent with correlational studies which suggest measures of trait mindfulness are associated with higher levels of life satisfaction [53–55]. Furthermore, sustained levels of PO and Life Satisfaction at follow up suggest that regular small practice with the class teacher following the training sessions may be required to maintain the increase in observed levels of mindfulness and wellbeing domains. Future research could build upon this by investigating how regular follow up practice at home may further improve the effectiveness of such an intervention [56] and whether the effect decreases following the six-week summer break. Finally, and in contrast to the other measures, the study failed to find any significant changes over time in PA, NA, or the SCWBS sub- scale of Positive Emotional State.

Given the Positive Outlook subscale (SCWBS) and the Life Satisfaction Scale (SLSS) tap into cognitive aspects of wellbeing, and in the absence of significant changes in scales representing affectivity (PA, NA and PeS), mindfulness may also enhance wellbeing through alternative cognitive mechanisms not exclusive to the regulation of emotions. In support of this suggestion, a study by Kong et al. [54] reported a mediating role of core self-evaluations in the link between life satisfaction and mindfulness. Indeed, within the context of children and mindfulness programmes, exploring this relationship further may be a potential avenue for future research.

The present study had many strengths. Sustained significant enhancements with medium effect sizes in both Positive Outlook and Life Satisfaction are promising, especially since the children only received a relatively modest ‘dosage’ of training. Medium effect sizes are also consistent with findings of a meta-analysis of school-based mindfulness programmes by Zenner et al. [31]. Results are also promising given the context of training being delivered by qualified class teachers and that additional home practice was not an explicit part of the programme. Moreover, the fact that the majority of prior studies concerning mindfulness in schools involved expert mindfulness trainers, the current study is more naturalistic by reflecting the realistic challenges schools face in implementing such programmes [38]. Significant findings of the present study are also promising given that they were obtained using stricter statistical analyses compared to other studies which adjusted for baseline imbalances (see e.g. [57]). Furthermore, the present study aimed to reduce bias by adopting separation between the developer and the researcher [7].

Despite these strengths the study also had its limitations. Due to class-based groups of children and volunteer availability, the study was unable to randomly allocate participants to each condition which somewhat reduces its power [58]. Alongside considerations of measurement sensitivity, this is a potential explanation for the lack of significant findings in the domains of Positive and Negative Affect for which the latter showed significant reductions at follow up in another UK based study [38]. However, random allocation was not achievable in the present study and as noted earlier, it can have its own limitations when conducted in the same school. As such, future studies in primary schools would benefit from using designs similar to the current large MYRIAD study which is evaluating the impact of mindfulness on adolescents by employing a parallel-group, cluster randomised controlled trial of 76 schools with a much longer 2 year follow up [59].

In attempts to gain further insight into the mechanisms of how mindfulness may enhance wellbeing, the study did not find any significant improvements in cognitive reappraisal despite suggestions mindfulness may strengthen and facilitate this strategy [60]. In the absence of significant changes across time, correlational analyses did however show that change scores in cognitive reappraisal were significantly positively correlated with mindfulness which were also associated with enhanced levels of Positive Outlook and Life Satisfaction. These findings are consistent with results of the study by Vickery and Dorjee [38] who found patterns of enhancement in emotional wellbeing between measures of emotional awareness, expression and mindfulness. Being correlational however, the directionality of this relationship is unknown. Consequently, future empirical studies are needed which specifically investigate the precise relationship between cognitive reappraisal, mindfulness and wellbeing.

It is at this point where consideration must be given to the measurement of emotion regulation and limitations of self-report methods per se. With questionable internal consistency of the ERQ-CA reappraisal subscale at pre and follow up, the suitability of self-report measures to effectively measure this construct is perhaps dubious. As such, the impact of mindfulness on emotion regulation may alternatively be investigated using mixed methods (including neuroscience contribution; e.g. [19]) which can explore the rich subjective personal responses of participants to the training and thus potentially counteract any weaknesses of both quantitative and qualitative methodologies [61]. Clearly, recent contribution from the neurosciences may also aid in the unravelling of the complex relationship that exists been mindfulness and emotion regulation [19]. Also, given other studies have indicated that cognitive reappraisal may enhance health by reducing the stress response to emotions and thus consequent cortisol secretion [60], future studies could also objectively measure salivary cortisol as a potential indicator of the ability of mindfulness to improve reappraisal. Despite greater internal consistency of other self-report measures used in the study, self-report measures are also vulnerable to response bias and interviewer effect [62]. In addition, children may respond in a socially desirable manner [63]. However, examination of the social desirability sub scale scores on the SCWBS indicated the children had not responded in this way. Finally, despite the procedure being designed to limit potential demand characteristics, in a similar fashion to cortisol for stress responses, future studies could overcome any threats to validity by using additional objective measures which reflect changes in subjective wellbeing such as heart rate variability [64].

In spite of issues with self-report methods, a major strength of the present study was the use of an active control which aimed to increase the validity of findings concerning the benefits of mindfulness and the obtained effect sizes being attributed to mindfulness alone. Without an active control, positive effects may have indeed been attributed to non-specific features of interventions such as adult contact time, possible expectation of benefits, time or novelty. The inclusion of an active control into the design was solely for this purpose however it was also designed to potentially have an impact on pupil wellbeing given results of previous positive psychological based school programmes (see e.g. [65]). Findings suggest that the active control had no significant effect on pupil wellbeing. Given the sample size however (*n* = 19), statistical power was particularly low and thus perhaps not large enough to detect a significant effect. However, the consideration of the abovementioned effect sizes (Table 3) are suggestive of benefits of the active control alongside notably enhanced effects in the mindfulness condition.

The acceptability data provided a useful starting point in understanding how mindfulness may more broadly benefit the children. Like previous studies (see e.g. [57]), high rates of acceptability were found. The survey also alluded to mindfulness having potential to enhance areas such as friendships, stress management, self-belief and confidence. These findings are congruent with results from a mixed methods study [37] and qualitative study [66] which indicated the positive impact of mindfulness on pupils’ wellbeing through the strengthening of social and emotional functioning. These findings are in line with the concept of the prosocial classroom model [67] in which supportive teacher student relationships alongside social and emotional learning may help improve behavioural and academic outcomes for pupils. As it was suggested earlier, future studies could further enrich these lines of enquiry by adopting a mixed methods approach.

Another limitation of the study was that it was unable to directly measure treatment fidelity of both the active control and mindfulness group. Although the fidelity of the mindfulness training was enhanced by stringent training requirements, future studies could potentially include supervision and feedback to staff from an experienced practitioner. Also, future studies could adopt implementation checks to improve adherence to the interventions [68]. Additionally, given that each class had a different member of staff delivering either mindfulness or the active control sessions, future studies could aim to take account of potential moderating factors such empathy, personality and motivation of staff. With this in mind, adoption of a multi-level analysis in future research may also prove useful given that data in the present study had a hierarchal structure [69]; wellbeing scores from pupils may be considered as being ‘nested’ within the staff who are themselves ‘nested’ in a particular school. Indeed, a meta-analysis reported many mindfulness studies rarely provide enough detail to account for nesting in their research design which in turn makes extracting exact effect sizes more problematic [35].

## Conclusions

In conclusion, within the context of an ever growing need to address pupil wellbeing in schools, this study showed that when delivered to a group of primary aged children, mindfulness training significantly enhanced levels of Positive Outlook and Life Satisfaction post training and at 3 months follow up. This study contributes to a research area in its infancy which is beginning to suggest school mindfulness programmes can be feasibly integrated into the curriculum as a universal and accessible approach to boosting pupil wellbeing and also a potential preventative approach to mental health. Given the relative lack of studies in this area, future studies should indeed replicate these findings. Additionally, due to threats to validity of self-report methods, more studies are required which incorporate objective data from physiological measures which may reflect changes in wellbeing. Finally, more research is required to understand the specific mechanisms by which mindfulness has its positive effect on wellbeing not just in the context of school training programmes, but in broader contexts of mindfulness practice.

## Data Availability

Data is available on request from the corresponding author.

## Abbreviations

AC: Active control
CAMHS: Child and Adolescent Mental Health Services
CAMM: The Child Adolescent Mindfulness Measure
ERQ-CA: Emotion regulation Questionnaire for Children and Adolescents (Cognitive Reappraisal)
LMPP: Living Mindfully Primary Programme
NHS: National Health Service
PANAS-C: The Positive and Negative Affect Scale for Children
PSHE: Personal, Social and Health Education
RCT: Randomised Controlled Trial
SCWB PeS: Sterling Children’s Wellbeing Scale (Positive Emotional State)
SCWBS PO: Sterling Children’s Wellbeing Scale (Positive Outlook)
SLSS: Student’s Life Satisfaction Scale
WLC: Wait list control
